# Mer-mediated eosinophil efferocytosis regulates resolution of allergic airway inflammation

**DOI:** 10.1016/j.jaci.2018.01.029

**Published:** 2018-12

**Authors:** Jennifer M. Felton, Christopher D. Lucas, David A. Dorward, Rodger Duffin, Tiina Kipari, Sonja Vermeren, Calum T. Robb, Kenneth G. MacLeod, Bryan Serrels, Jürgen Schwarze, Christopher Haslett, Ian Dransfield, Adriano G. Rossi

**Affiliations:** a MRC Centre for Inflammation Research, the Queen's Medical Research Institute, University of Edinburgh, Edinburgh, United Kingdom; b MRC Institute of Genetics and Molecular Medicine, University of Edinburgh, Western General Hospital Campus, Edinburgh, United Kingdom

**Keywords:** Eosinophil, apoptosis, phagocytosis, Mer, inflammation resolution, allergic airway inflammation, airway resistance, AM, Alveolar macrophage, BALF, Bronchoalveolar lavage fluid, BMDM, Bone marrow–derived macrophage, bmEos, Bone marrow–derived eosinophils, DMEM, Dulbecco modified Eagle medium, FCS, Forward scatter, IMDM, Iscove modified Dulbecco medium, Mer^KD^, Mer-deficient/kinase dead, OVA, Ovalbumin, Pros1, Protein S, SSC, Side scatter, TAM, Tyro-3/Axl/Mer, WT, Wild type

## Abstract

**Background:**

Eosinophils play a central role in propagation of allergic diseases, including asthma. Both recruitment and retention of eosinophils regulate pulmonary eosinophilia, but the question of whether alterations in apoptotic cell clearance by phagocytes contributes directly to resolution of allergic airway inflammation remains unexplored.

**Objectives:**

In this study we investigated the role of the receptor tyrosine kinase Mer in mediating apoptotic eosinophil clearance and allergic airway inflammation resolution *in vivo* to establish whether apoptotic cell clearance directly affects the resolution of allergic airway inflammation.

**Methods:**

Alveolar and bone marrow macrophages were used to study Mer-mediated phagocytosis of apoptotic eosinophils. Allergic airway inflammation resolution was modeled in mice by using ovalbumin. Fluorescently labeled apoptotic cells were administered intratracheally or eosinophil apoptosis was driven by administration of dexamethasone to determine apoptotic cell clearance *in vivo*.

**Results:**

Inhibition or absence of Mer impaired phagocytosis of apoptotic human and mouse eosinophils by macrophages. Mer-deficient mice showed delayed resolution of ovalbumin-induced allergic airway inflammation, together with increased airway responsiveness to aerosolized methacholine, increased bronchoalveolar lavage fluid protein levels, altered cytokine production, and an excess of uncleared dying eosinophils after dexamethasone treatment. Alveolar macrophage phagocytosis was significantly Mer dependent, with the absence of Mer attenuating apoptotic cell clearance *in vivo* to enhance inflammation in response to apoptotic cells.

**Conclusions:**

We demonstrate that Mer-mediated apoptotic cell clearance by phagocytes contributes to resolution of allergic airway inflammation, suggesting that augmenting apoptotic cell clearance is a potential therapeutic strategy for treating allergic airway inflammation.

Eosinophils play a major role in the propagation of allergic airway diseases, such as asthma.^1^, ^2^ During inflammation, eosinophils are recruited from the bone marrow and migrate to inflamed tissue, where they can release a range of cytotoxic eosinophil-derived products that promote inflammation, tissue remodeling, airway hyperresponsiveness, and organ dysfunction.^3^

Tissue presence of eosinophils is determined based on both recruitment and retention within inflamed sites. Eosinophil elimination from the lung can be regulated by transepithelial migration and mucociliary clearance or by apoptosis and subsequent phagocytosis by macrophages, dendritic cells, and airway epithelial cells, a process termed efferocytosis.^4^ The relative role and importance of eosinophil apoptosis and efferocytosis in the resolution of allergic airway inflammation in human subjects remains controversial,^5^ but several lines of evidence suggest that these pathways have relevance to allergic disease states. Prolonged eosinophil longevity (with reduced apoptosis) is associated with increasing asthma severity in human subjects,^6^ whereas macrophages from patients with severe or poorly controlled asthma have defective efferocytosis.^7^, ^8^ In addition, we have shown recently that driving eosinophil apoptosis with the flavone wogonin attenuates allergic lung inflammation in mice *in vivo*,^9^ suggesting that modulation of eosinophil apoptosis is a *bona fide* target for treating allergic diseases. The question of whether alterations in apoptotic cell clearance by phagocytes contributes directly to resolution of allergic airway inflammation remains to be addressed.

Although the molecular mechanisms driving changes in eosinophil lifespan and clearance *in vivo* remain poorly defined, it is known that glucocorticoids, the main treatment for asthma and other allergic diseases, induce eosinophil apoptosis and upregulate macrophage phagocytosis of apoptotic cells *in vitro*.^10^, ^11^ Glucocorticoid-augmented efferocytosis is dependent on Mer,^11^ a member of the Tyro-3/Axl/Mer (TAM) receptor tyrosine kinase family.^12^ There are 2 well-defined ligands for Mer, protein S (Pros1) and growth arrest-specific 6, which can bridge to phosphatidylserine exposed on apoptotic cells. The importance of TAM receptors and their ligands in efferocytosis has been demonstrated by using Mer-deficient/kinase dead (Mer^KD^) and triple TAM-deficient mice. These mice are characterized by impaired efferocytosis in lymphoid tissues, diminished apoptotic germ cell removal by Sertoli cells in the testis, and defective pruning of the photoreceptors in the retina by retinal pigment epithelial cells.^13^, ^14^, ^15^ Previous studies have investigated the role of Mer in neutrophil-dominant lung injury models (induced by LPS and bleomycin),^16^ whereas Axl downregulation has been demonstrated in patients with moderate-to-severe asthma.^17^ However, the potential role of Mer in regulating eosinophil clearance and resolution of allergic airway inflammation remains unexplored.

In the present study we investigated Mer-mediated eosinophil efferocytosis and its role in allergic airway inflammation resolution *in vivo* to establish whether apoptotic cell clearance directly affects the resolution of allergic airway inflammation. Absence or inhibition of Mer impaired phagocytosis of apoptotic human and mouse eosinophils by macrophages, whereas Mer-deficient mice had delayed resolution of ovalbumin (OVA)–induced allergic airway inflammation.

## Methods

### Eosinophil isolation

Human granulocytes were isolated from blood of healthy volunteers, as previously described (Lothian Research Ethics Committee, #08/S1103/38; #15-HV-013).^18^ Eosinophils were subsequently isolated by using anti-CD16^+^ microbeads (#130-045-701; Miltenyi Biotec, Bergisch Gladbach, Germany), according to the manufacturer's instructions, with a purity of greater than 95%, as assessed by using cellular morphology of Diff-Quik–stained cytocentrifuge preparations. Cells were cultured in Iscove modified Dulbecco medium (IMDM; Gibco, Carlsbad, Calif) with 10% autologous serum (37°C in a 5% CO_2_ atmosphere).

Mouse bone marrow–derived eosinophils (bmEos) were generated from unselected bone marrow progenitor cells by using an extended 14-day version of a described protocol.^19^ Briefly, bone marrow cells were cultured in RPMI 1640 supplemented with 20% FCS, 100 IU/mL penicillin/streptomycin, 2 mmol/L l-glutamine, 25 mmol/L HEPES (Sigma, St Louis, Mo), 1× nonessential amino acids, and 1 mmol/L sodium pyruvate (both from Gibco), 50 μmol/L 2-mercaptoethanol, stem cell factor (100 ng/mL; PeproTech, Rocky Hill, NJ) and FLT3 ligand (100 ng/mL; PeproTech) for the first 4 days before switching to media containing IL-5 (10 mg/mL; PeproTech) for the remainder of the culture period. After 14 days, cells were more than 95% eosinophils, as assessed based on cellular morphology and expression of Siglec-F determined by using flow cytometry.

### Macrophage isolation

Mouse bone marrow–derived macrophages (BMDMs) were generated, as previously described.^20^ Tibias and femurs were flushed with Dulbecco modified Eagle medium (DMEM; Gibco) and red blood cells were lysed with ACK lysis buffer (Gibco) before passing through a 40-μm cell strainer. Cells were plated onto 15-cm cell-culture dishes (Corning, Corning, NY) in DMEM with 20% FCS, 100 IU/mL penicillin/streptomycin, and 20% L929 supernatant. Media were replaced after 3 days. On day 6, differentiated macrophages were washed in PBS (Gibco) and detached with a cell scraper. Cells were plated at 0.7 × 10^6^/mL in DMEM without serum for 1 hour to allow adhesion before culturing in DMEM with 10% FCS with or without 200 nmol/L dexamethasone for 24 hours before experimentation, a widely established protocol to enhance efferocytosis.^12^, ^21^

Mouse alveolar macrophages (AMs) were obtained by means of lung lavage with 10 mL of PBS/0.5 mmol/L EDTA. AMs were centrifuged at 350*g* for 5 minutes, resuspended in IMDM, and incubated at 150,000 cells per well in a 96-well plate. After 1 hour, culture media were replaced with IMDM supplemented with 10% FCS before overnight incubation.

### *In vitro* phagocytosis assays

Analysis of phagocytosis of fluorescently labeled apoptotic cells was performed by using a modification of a previously described method.^12^ Macrophages were stained with CellTrace Far Red (Thermo Fisher Scientific, Waltham, Mass), according to the manufacturer's instructions, before addition of apoptotic cells. Human eosinophil constitutive apoptosis was induced by means of overnight culture, whereas apoptosis of mouse bmEos was induced by overnight culture with 1 μmol/L budesonide in the absence of IL-5. Apoptosis was examined by using Annexin V and propidium iodide staining with flow cytometry. Apoptotic eosinophils were labeled with pHrodo, according to the manufacturer's instructions, and then washed and resuspended at 4 × 10^6^/mL (human eosinophils) or 5 × 10^6^ (mouse bmEos) in IMDM and coincubated with macrophages for 1 hour with 33 nmol/L Pros1 with or without 1 μmol/L BMS777607 (Selleck Chemicals, Houston, Tex), according to the figure legends. After coincubation, macrophages were detached with 0.05% trypsin/0.53 mmol/L EDTA, and phagocytosis was assessed by using flow cytometry (BD LSR Fortessa; BD Biosciences, San Jose, Calif).^22^, ^23^

### Western blotting

Western blotting was performed, as previously described.^24^, ^25^ Briefly, BMDMs were lysed in 0.1% Nonidet P40 containing a protease inhibitor cocktail.^25^ Lysates were separated on a 12% Tris-HEPES Precise gel (Thermo Fisher Scientific) and transferred electrophoretically onto polyvinylidene difluoride membranes (Merck Millipore, Burlington, Mass). Membranes were blocked with 5% nonfat milk (Marvel) in Tris-buffered saline/0.1% Tween-20 before incubation with primary antibodies directed against Mer (1:000; AF591; R&D Systems, Minneapolis, Minn) and β-actin (1:50,000; A1978, Sigma). This was followed by horseradish peroxidase–conjugated secondary antibodies (1:2500; Dako, Glostrup, Denmark) and incubation with ECL prime (GE Healthcare, Fairfield, Conn). Blots were exposed to light-sensitive film (MOL7016; Scientific Laboratory Supplies, Nottingham, United Kingdom) and processed through an X-ray developer (Ecomax Processor; Photo Imaging Systems, Germany).

### *In vivo* model of allergic airway inflammation

Experiments were performed in accordance with the UK Home Office Animals (Scientific Procedures) Act of 1996 after review by a local ethics committee. Wild-type (WT) control (C57BL/6; Charles River Laboratories, Wilmington, Mass) and Mer^KD^ mice (C57BL/6 background)^26^ were bred and maintained in specific pathogen-free conditions. Genotypes were confirmed before experimental procedures, with 6- to 8-week-old mice used for *in vitro* experiments and 8- to 16-week-old female mice used for *in vivo* experiments. OVA-induced allergic airway inflammation was modeled, as described previously.^9^ Briefly, mice were sensitized by means of intraperitoneal alum-precipitated (Alum Imject; Pierce Biotechnology, Waltham, Mass) OVA (20 μg of OVA and 50 μL of alum per mouse; Sigma) on days 1 and 10 and challenged on days 22, 23, and 24 by means of intratracheal OVA (50 μg). Mice were culled, and bronchoalveolar lavage fluid (BALF) and lung tissue were acquired and processed, as previously described.^18^

BALF cells and lung interstitial inflammatory cells were incubated with combinations of antibodies against CD45, CD11b, Ly6G, Siglec-F, and F4/80, with flow cytometric analysis performed in the presence of Flow-Check Fluorosphere counting beads (Beckman Coulter, Fullerton, Calif) to allow quantification of cell numbers. The resolution interval (the time for eosinophil numbers to decrease to half-maximal numbers) was calculated, as previously described.^27^ BALF cytokines and mucus (MUC5AC) were quantified by means of ELISA (R&D Systems or Caltag Medsystems, Buckingham, United Kingdom) or by means of forward-phase protein microarray and expressed as either relative to lavage fluid control (PBS) or as relative expression in Mer^KD^ mice compared with WT control mice at day 7 after OVA challenge (as indicated in the figure legends).

Lungs from separate animals were fixed in 10% formalin (Sigma) before sectioning and staining with hematoxylin and eosin or periodic acid–Schiff. Histologic scoring of alveolar and interstitial inflammatory cell infiltration was quantified after analysis by 2 independent blind observers, with quantification of airway mucus production performed by using the mucus goblet index, as previously described.^9^

Airway responsiveness to aerosolized methacholine was assessed in anesthetized and mechanically ventilated WT and Mer^KD^ mice after sensitization and challenge with OVA (Buxco Research Systems, Wilmington, NC). Lung resistance was determined and expressed relative to baseline values in the absence of methacholine with nebulized PBS administered as a vehicle. In the absence of methacholine challenge, airway resistance was measured in OVA-naive WT and Mer^KD^ mice.

In separate experiments dexamethasone (2 mg/kg) was administered intraperitoneally on day 3 after OVA to induce eosinophil apoptosis before quantification of BALF apoptotic eosinophil numbers by Annexin V binding (of CD45^+^CD11b^+^Ly6G^−^Siglec-F^+^ cells).

### *In vivo* phagocytosis experiments

After isolation from peripheral blood, human granulocyte apoptosis was induced by overnight culture before labeling with CellTracker Green, according to the manufacturer's instructions. Apoptosis was confirmed by using Annexin V and propidium iodide staining with flow cytometry. A total of 100,000 labeled apoptotic human cells were administered intratracheally to naive WT and Mer^KD^ mice. Mice were culled after 0 and 3 hours, and BALF was retrieved for analysis of CellTracker Green–positive AMs (CD45^+^CD11c^+^CD11b^−^ cells), uncleared apoptotic cells (mouse CD45^−^CellTracker Green^+^ cells), recruited mouse granulocytes (CD45^+^Ly6G^+^ cells), and necrotic debris (epithelial cell adhesion molecule–negative, CD11c^−^F4/80^−^, low side-scatter [SSC]/forward-scatter [FSC] events).

### Data analysis

Data were analyzed with GraphPad Prism software (v5; GraphPad Software, La Jolla, Calif), with flow cytometric data analyzed by using FlowJo software (TreeStar, Ashland, Ore). All data are expressed as means ± SEMs and analyzed by using the Student *t* test or ANOVA as appropriate, with significance accepted at a *P* value of less than .05.

## Results

### Mer deficiency delays resolution of allergic airway inflammation

**Fig 1:**
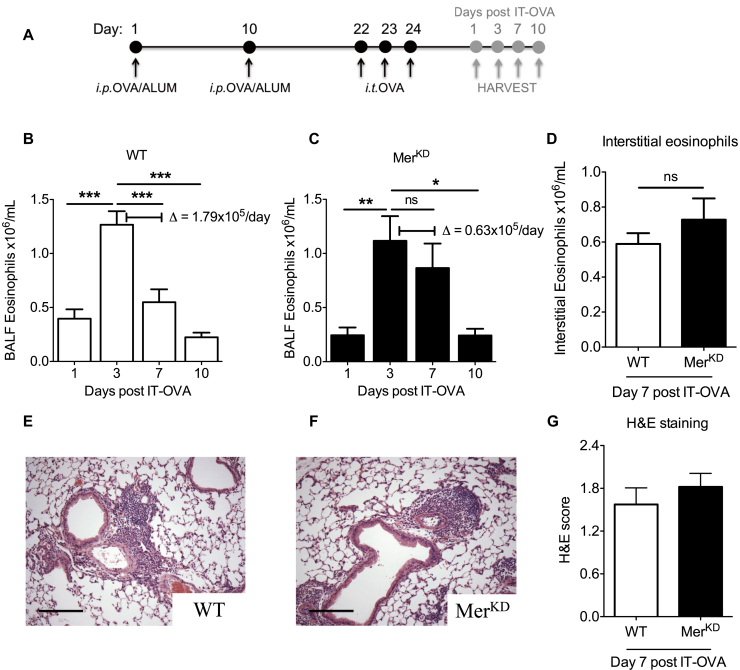
Mer^KD^ mice have delayed resolution of allergic airway inflammation *in vivo.***A,** Schema of the experimental protocol. *i.p.*, Intraperitoneal. **B** and **C,** BALF eosinophils in WT (Fig 1, *B*) and Mer^KD^ (Fig 1, *C*) mice at 1, 3, 7, and 10 days after OVA, with Δ change in eosinophil numbers between day 3 and day 7 shown (n = 7-10). **D,** Interstitial eosinophils at day 7 in WT and Mer^KD^ mice (n = 6-8). **E** and **F,** Representative lung sections stained with hematoxylin and eosin at day 7 after OVA from WT (Fig 1, *E*) and Mer^KD^ (Fig 1, *F*) mice (×100 original magnification). *Scale bar* = 20 μm. **G,** Quantification of hematoxylin and eosin–stained lung sections at 7 days after OVA treatment (n = 5). Data are expressed as means ± SEMs and analyzed by means of 1-way ANOVA with the Newman-Keuls multiple comparison test (Fig 1, *B* and *C*) or the Student *t* test (Fig 1, *D* and *G*), *P < .05, **P < .01, and ***P < .001. *IT-OVA*, Intratracheal ovalbumin; *ns*, not significant.

WT and Mer-deficient (Mer^KD^) mice were sensitized and challenged with OVA before acquisition of tissue on days 1, 3, 7, and 10 after OVA challenge to investigate the role of apoptotic cell clearance in the resolution of allergic airway inflammation (Fig 1, *A*). Although both WT and Mer^KD^ mice had similar peak BALF eosinophil numbers at day 3 after OVA (1.27 ± 0.12 vs 1.12 ± 0.23 × 10^6^/mL), delayed resolution of eosinophilic inflammation was observed in the Mer^KD^ mice at day 7 (Fig 1, *B* and *C*). This revealed a Δ change in BALF eosinophil numbers between day 3 and day 7 of 1.79 × 10^5^ cells/d in the WT mice versus 0.63 × 10^5^ cells/d in the Mer^KD^ mice, with a resolution interval that was prolonged by 2 days in the Mer^KD^ mice (6.5 vs 8.5 days). Interestingly, numbers of interstitial eosinophils were similar at day 7 (0.58 ± 0.06 vs 0.73 ± 0.12 × 10^6^ cells/mL; Fig 1, *D*). Similarly, hematoxylin and eosin–stained lung sections demonstrated predominantly perivascular inflammation, with no significant differences seen between WT and Mer^KD^ mice (Fig 1, *E-G*).

**Fig 2:**
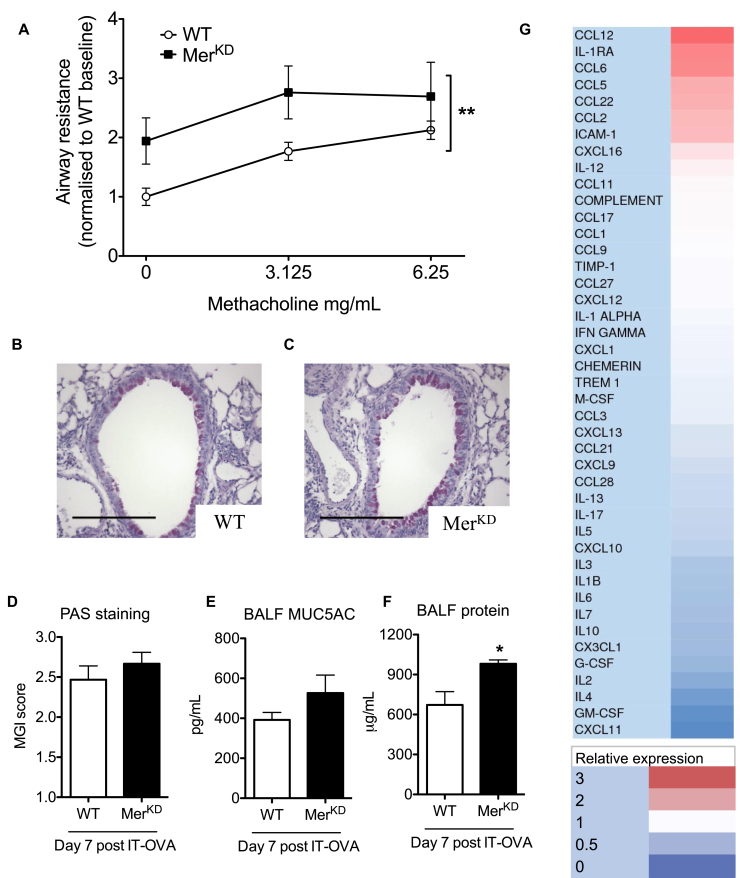
Mer^KD^ mice have exacerbated allergic airway responses. **A,** Airway responsiveness to aerosolized methacholine was assessed in anesthetized and mechanically ventilated mice at 7 days after OVA, with lung resistance expressed relative to WT baseline (after nebulization of PBS without methacholine, n = 4-5). **B** and **C,** Representative day 7 lung sections stained with periodic acid–Schiff (PAS) from WT (Fig 2, *B*) and Mer^KD^ (Fig 2, *C*) mice (×200 original magnification). **D,** Quantification of mucus production at day 7, as assessed by using the mucus-goblet index *(MGI)* on PAS-stained lung tissue sections (n = 5). **E** and **F,** BALF Mucin5AC (a mucus glycoprotein; Fig 2, *E*) and total protein content (Fig 2, *F*) were measured at day 7 after OVA (n = 6-8). **G,** Cytokine array showing day 7 cytokines, chemokines, and proteins upregulated in the Mer^KD^ mice depicted in red and downregulated depicted in blue. *IT-OVA*, Intratracheal ovalbumin. Data are expressed as means ± SEMs and analyzed by means of 2-way ANOVA (Fig 2, *A*) or the Student *t* test (Fig 2, *D-F*). *P < .05 and **P < .01.

**Fig E1:**
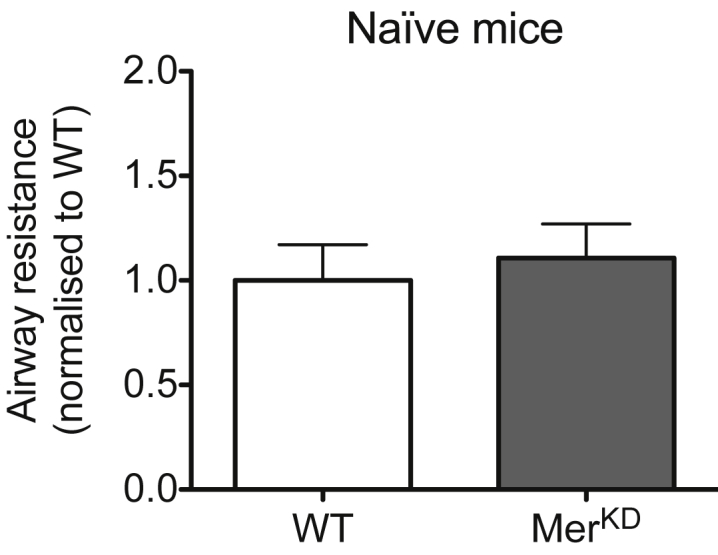
Lack of Mer has no effect on airway resistance in naive mice. Relative airway resistance was assessed in anesthetized and mechanically ventilated naive WT and Mer^KD^ mice in the absence of methacholine and expressed relative to WT mouse values. Data are expressed as means ± SEMs and analyzed by using the Student *t* test (n = 6-7).

The functional consequences of the delayed inflammation resolution in Mer^KD^ mice were explored by measuring airway responsiveness in anesthetized and mechanically ventilated mice in response to aerosolized methacholine. Mer^KD^ mice that had been sensitized and challenged with OVA had increased airway resistance (Fig 2, *A*). Baseline airway resistance (in the absence of methacholine) was unaltered in naive Mer^KD^ mice (see Fig E1 in this article's Online Repository at www.jacionline.org), confirming that the observed increase in airway resistance was not innate but specific to the presence of allergic airway inflammation. Airway mucus production was similar in both WT and Mer^KD^ mice (Fig 2, *B-E*), although BALF total protein levels were increased in Mer^KD^ mice (671.7 ± 99.9 vs 990.0 ± 28.8 μg/mL, *P* < .05), which is consistent with the increased inflammation observed (Fig 2, *F*).

### Enhanced cytokine production is not a major feature of allergic inflammation in Mer^KD^ mice

**Fig E2:**
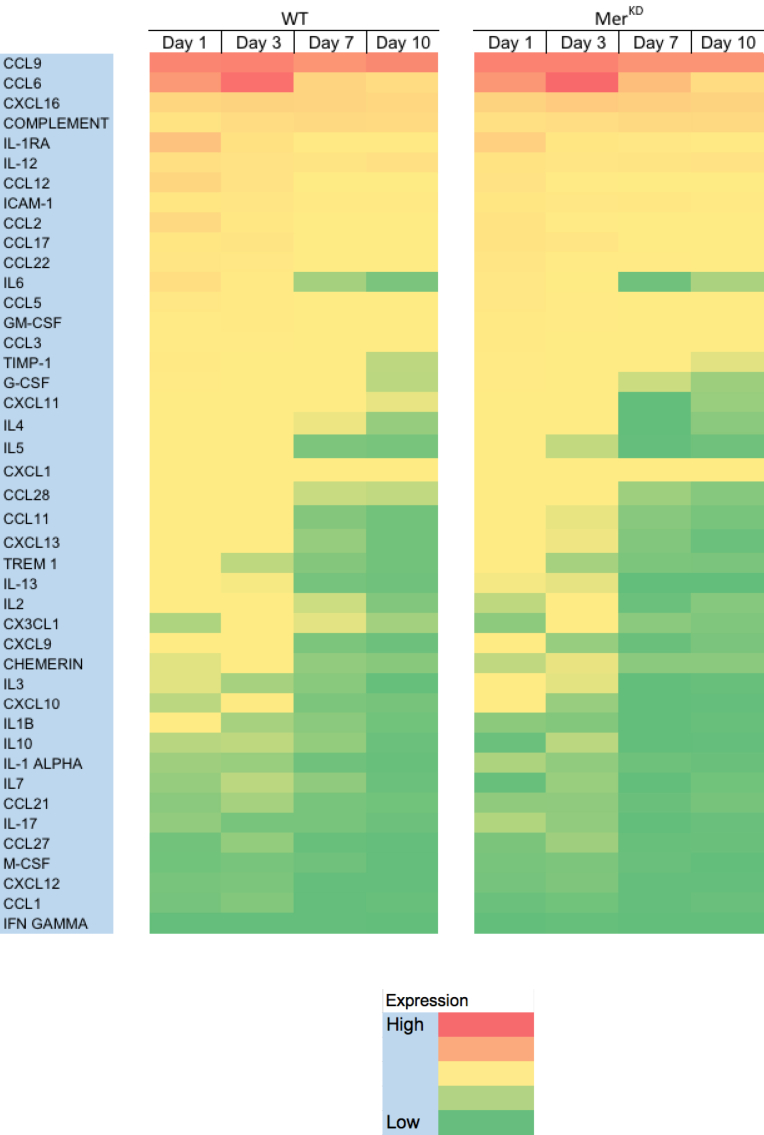
Time course of BALF cytokines and proteins in WT and Mer^KD^ mice after OVA. BALF cytokine and protein levels were measured by using the forward-phase protein array (n ≥ 3 per genotype at each time point), with red depicting high expression and green depicting low expression.

Given that signaling through TAM receptors, including Mer, acts to suppress proinflammatory cytokine production,^28^ we analyzed BALF cytokines to examine whether increased cytokine levels were present in Mer^KD^ mice and contribute to excess inflammation. Alterations in BALF protein and cytokine levels were investigated by means of analysis of 43 separate targets at 4 separate time points. This analysis revealed that the overall pattern of cytokine expression was similar between Mer^KD^ and WT mice (see Fig E2 in this article's Online Repository at www.jacionline.org), with 8 cytokines upregulated more than 10% and 21 downregulated more than 10% in the Mer^KD^ mice at day 7 (Fig 2, *G*). Despite increased relative expression of monocyte chemoattractant protein 5 (CCL12), RANTES (CCL5), and monocyte chemoattractant protein 1 (CCL2) in the BALF of Mer^KD^ mice at day 7, these chemokines/cytokines were expressed at low absolute levels (data not shown). Overall, we interpret these data as suggesting that enhanced cytokine production was not the major mechanism behind the delayed resolution of allergic inflammation observed in Mer^KD^ mice.

### Mer inhibition or deficiency impairs phagocytosis of apoptotic eosinophils

**Fig 3:**
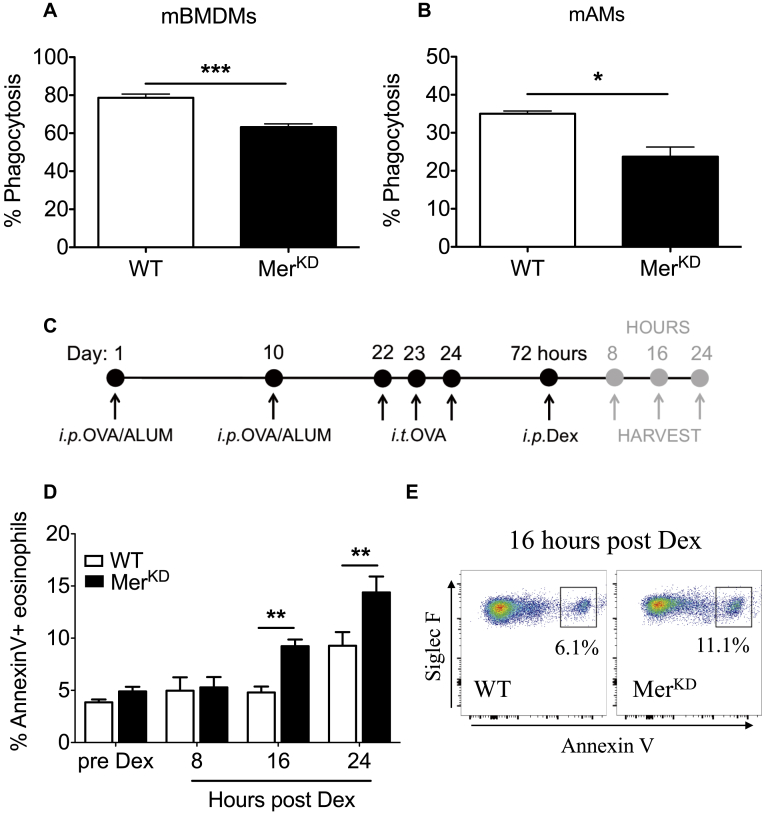
Mer deficiency impairs phagocytosis of apoptotic eosinophils. **A,** Phagocytic capacity of mouse BMDMs *(mBMDMs)* from WT or Mer^KD^ mice was assessed after coculture with apoptotic mouse bmEos in the presence of Pros1 (n = 4-5). **B,** Phagocytic capacity of mouse AMs *(mAMs)* from WT or Mer^KD^ mice was assessed after coculture with apoptotic human eosinophils in the presence of Pros1 (n = 4-5). **C,** Schema of *in vivo* experimental protocol. *i.p.*, Intraperitoneal; *i.t.*, intratracheal. **D,** Annexin V binding of BALF eosinophils from WT or Mer^KD^ OVA-treated mice at 8, 16, and 24 hours after dexamethasone *(Dex)* treatment (n = 4-7). **E,** Representative flow cytometric plots (Annexin V/Siglec-F) from WT and Mer^KD^ mice showing the presence of Annexin V^+^ eosinophils (CD45^+^CD11b^+^Ly6G^−^Siglec-F^+^Annexin V^+^ cells) at 16 hours after dexamethasone. Data are expressed as means ± SEMs and analyzed by using the Student *t* test (Fig 3, *A* and *B*) or 2-way ANOVA with the Bonferroni test (Fig 3, *D*). *P < .05, **P < .01, and ***P < .001.

**Fig E3:**
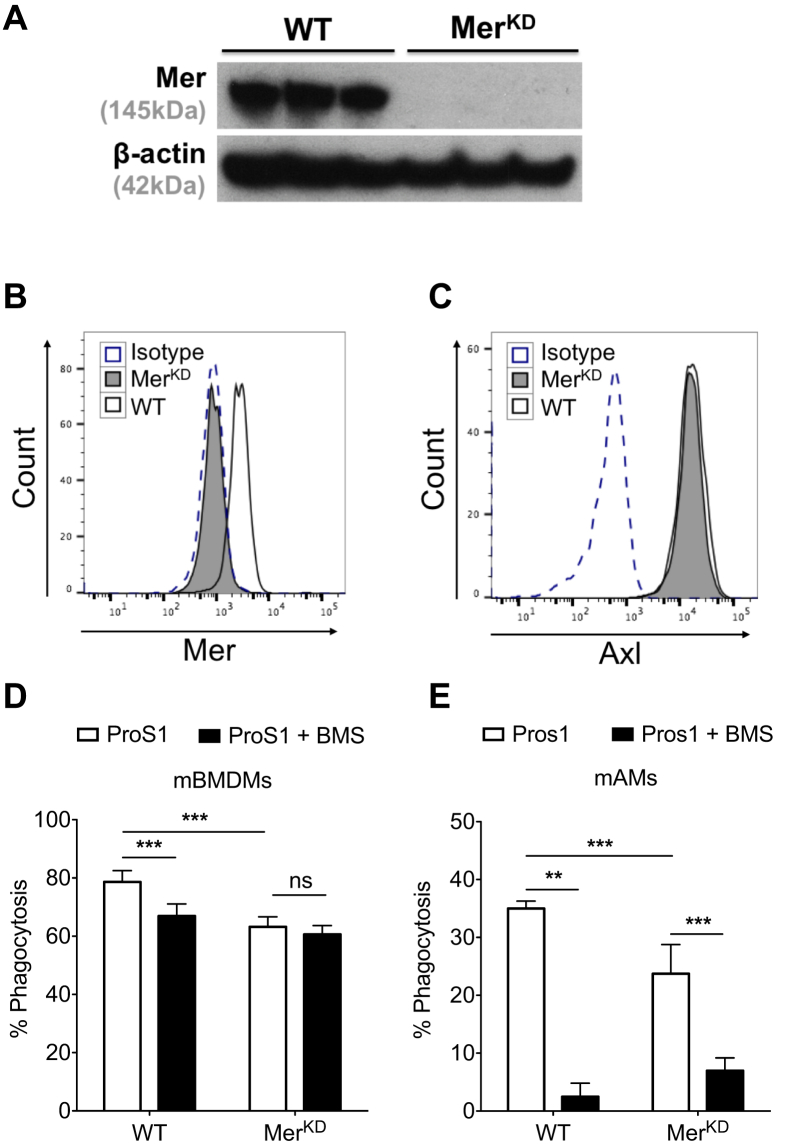
Confirmation of Mer expression on BMDMs and AMs from WT mice and absent expression in Mer^KD^ mice. **A,** Western blot of lysates of BMDMs from WT or Mer^KD^ mice. **B** and **C,** Representative flow cytometric histograms of AMs isolated from WT and Mer^KD^ mice showing Mer (Fig E3, *B*) and Axl (Fig E3, *C*) expression. **D,** Mouse BMDMs *(mBMDMs)* from WT or Mer^KD^ mice were cocultured with apoptotic mouse bmEos in the presence of Pros1 with or without BMS777607 (*BMS*; c-met inhibitor to inhibit Axl/Tyro3/Mer; n = 4-5). **E,** Mouse AMs *(mAMs)* from WT or Mer^KD^ mice were cocultured with apoptotic human eosinophils in the presence of Pros1 with or without BMS777607 (n = 4-5). Data are expressed as means ± SEMs and analyzed by using 2-way ANOVA with the Bonferroni test (Fig E3, *E* and *F*). **P < .01 and ***P < .001. *ns*, Not significant.

**Fig E4:**
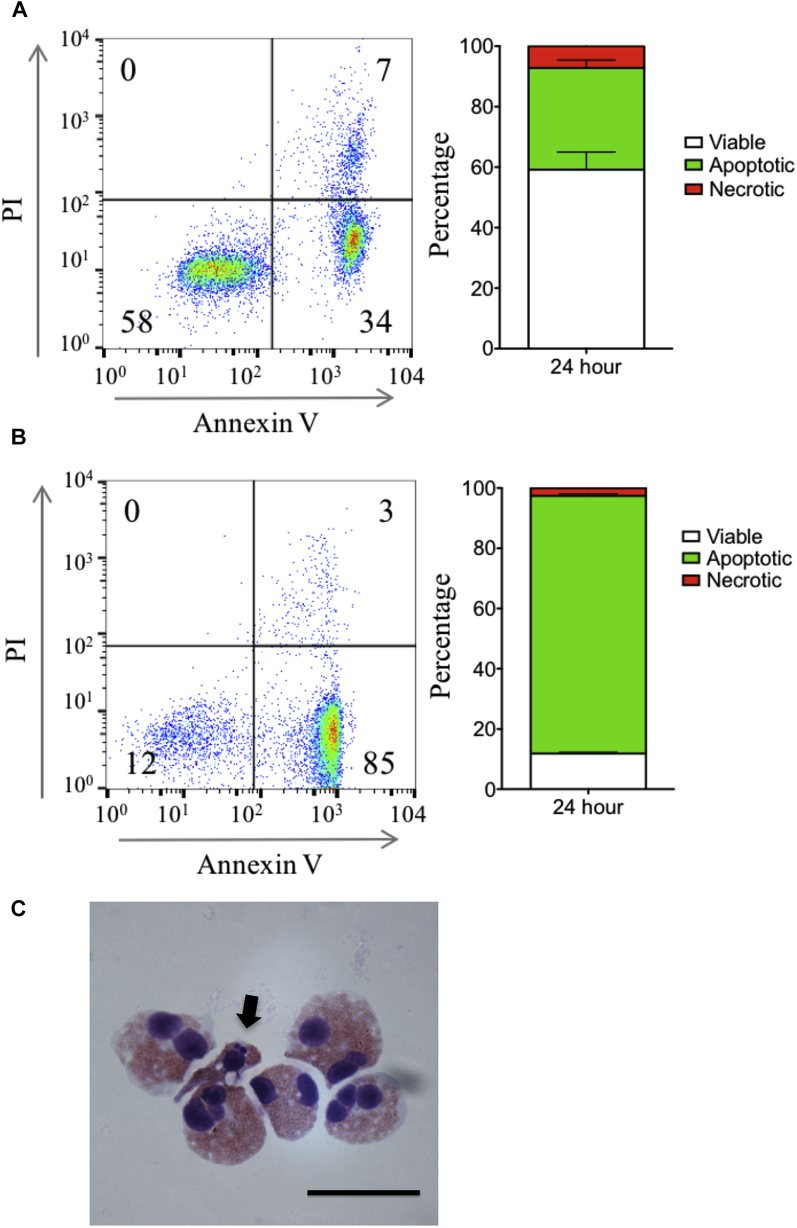
Analysis of human granulocyte viability, apoptosis, and necrosis. **A** and **B,** Assessment of viable (Annexin V^−^ propidium iodide *[PI]*^−^), apoptotic (Annexin V^+^PI^−^), and necrotic (PI^+^) human eosinophils, as determined by using Annexin V/PI staining with flow cytometry, before incubation with mouse AMs (Fig E4, *A*) and human granulocytes (Fig E4, *B*) undergoing constitutive apoptosis before intratracheal administration to mice is shown. Example flow cytometric plots and cumulative data are shown (n = 2-4). **C,** Representative cytocentrifuge preparation of aged human eosinophils. *Black arrow* highlights an apoptotic eosinophil with typical cellular shrinkage and nuclear condensation (×1000 original magnification). *Scale bar* = 20 μm.

We next investigated the relevance of Mer-mediated apoptotic eosinophil clearance to the observed delayed resolution of allergic airway inflammation in Mer^KD^ mice. Loss of Mer expression on macrophages from Mer^KD^ mice was confirmed by means of Western blotting of BMDMs and by using flow cytometry of AMs (see Fig E3 in this article's Online Repository at www.jacionline.org). WT AMs expressed both Mer and Axl, with Axl expression unchanged on AMs from Mer^KD^ mice (see Fig E3, *B* and *C*). BMDMs (Fig 3, *A*) and AMs (Fig 3, *B*) from WT and Mer^KD^ mice were cocultured with pHrodo-labeled apoptotic eosinophils to assess macrophage capacity for efferocytosis (see Fig E4 in this article's Online Repository at www.jacionline.org). Exogenous Mer ligand (Pros1) was also added to these experiments to ensure that Mer-dependent efferocytosis was not limited by bridging ligand availability. Although we observed a significant component of Mer-independent phagocytosis of apoptotic eosinophils, around 30% of AM phagocytosis was Mer dependent (Fig 3, *A* and *B*). Furthermore, AMs treated with BMS777607 (a c-Met inhibitor that inhibits Axl, Tyro3, and Mer) displayed substantial inhibition of phagocytosis of apoptotic eosinophils (see Fig E3, *D* and *E*), which is consistent with expression of both Mer and Axl by AMs. Inhibition of BMDM efferocytosis by BMS777607 was less marked, which is consistent with low-level expression of Axl by these cells (data not shown) and previous data demonstrating that WT and Axl^−/−^ BMDMs have similar rates of efferocytosis.^12^

### Mer augments apoptotic cell clearance *in vivo* to dampen inflammation

**Fig E5:**
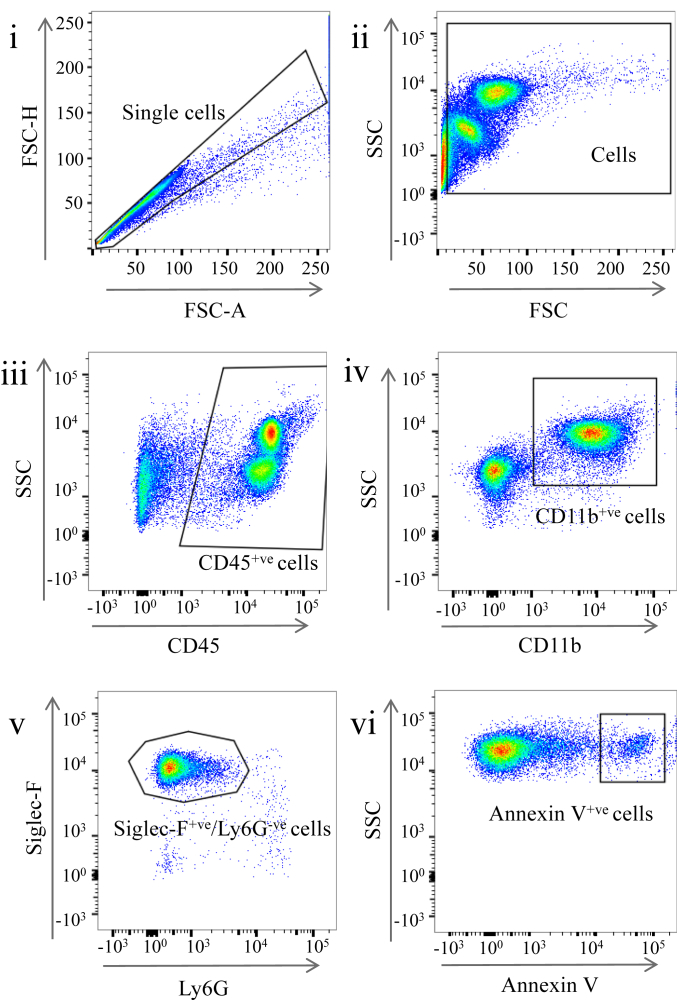
Flow cytometric gating strategy to identify Annexin V^+^ eosinophils. Representative flow cytometric plots showing the gating strategy used to identify Annexin V^+^ eosinophils (CD45^+^CD11b^+^Siglec-F^+^Ly6G^−^Annexin V^+^ cells) within BALF of OVA-challenged mice.

To further investigate the role of Mer-mediated engulfment of apoptotic eosinophils *in vivo*, the glucocorticoid dexamethasone was administered to OVA-sensitized and challenged mice at the peak of inflammation (day 3 after OVA) to induce eosinophil apoptosis (Fig 3, *C*).^10^ BALF was acquired at 8, 16, and 24 hours after dexamethasone administration, and eosinophils were analyzed for evidence of cellular death by using Annexin V binding (see Fig E5 in this article's Online Repository at www.jacionline.org). This revealed a time-dependent increase in the percentage of Annexin V^+^ eosinophils seen in BALF of Mer^KD^ mice (4.8% ± 0.5% vs 9.2% ± 0.6 at 16 hours, *P* < .05; Fig 3, *D* and *E*), which is consistent with a compromised capacity for eosinophil clearance in the absence of Mer.

**Fig 4:**
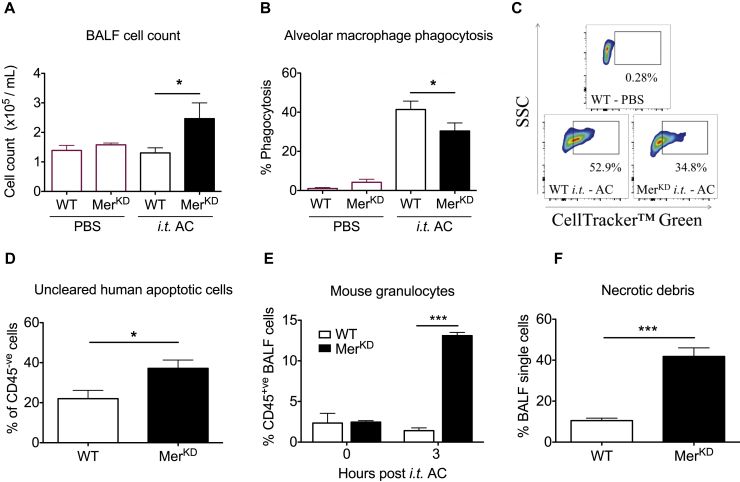
Mer^KD^ AMs have an impaired engulfment capacity, causing delayed apoptotic cell clearance *in vivo.* CellTracker Green fluorescently labeled apoptotic human cells *(AC)* or PBS controls were administered intratracheally *(i.t.)* to naive WT and Mer^KD^ mice, with BALF collected after 3 hours. **A-C,** Total BALF cell count (Fig 4, *A*), percentage of AMs (CD45^+^CD11c^+^CD11b^−^) phagocytosing labeled apoptotic cells (Fig 4, *B*), and representative flow cytometric plots (SSC/CellTracker Green; Fig 4, *C*) from WT-PBS or intratracheal AC treated WT and Mer^KD^ mice showing CellTracker Green^+^ (ie, engulfing) AMs (n = 3 WT-PBS; n = 6-7 intratracheal AC treated WT and Mer^KD^ mice). **D,** Uncleared apoptotic cells (CD45^−^CellTracker Green^+^ cells) in BALF after 3 hours (n = 7-9). **E,** Recruited mouse granulocytes (CD45^+^Ly6G^+^ cells) in BALF (n = 2-3). **F,** Percentage of necrotic debris present in BALF 3 hours after apoptotic cell administration (n = 8-10). Data are expressed as means ± SEMs and analyzed by using the Student *t* test. *P < .05 and ***P < .001.

**Fig E6:**
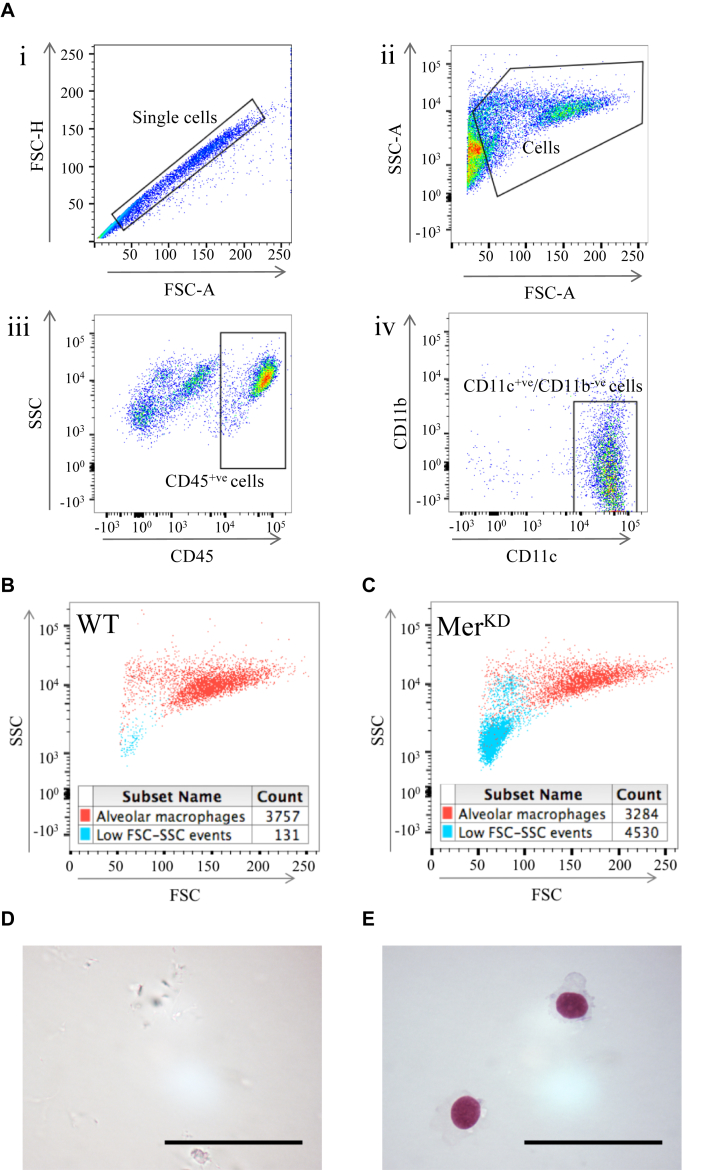
Identification of AM phagocytosis of apoptotic cells, uncleared human granulocytes, and necrotic debris *in vivo*. **A,** Representative flow cytometric plots showing the gating strategy used to identify AMs (CD45^+^CD11c^+^CD11b^−^ cells) within the BALF of apoptotic cell–treated mice. **B** and **C,** Representative flow cytometric plots demonstrating the AM population (red) and low-FSC/SSC necrotic debris population (blue) in BALF from WT (Fig E6, *B*) and Mer^KD^ (Fig E6, *C*) mice 3 hours after administration of apoptotic cells. **D** and **E,** Cytocentrifuge preparations of low-FSC/SSC events (Fig E6, *D*) and CD45^+^F4/80^+^CD11c^+^ AMs (Fig E6, *E*) sorted by means of fluorescence-activated cell sorting (×1000 original magnification). *Scale bar* = 40 μm.

Furthermore, direct intratracheal administration of labeled human granulocytes that had undergone cell death (predominantly apoptosis; see Fig E4, *B*) to naive WT and Mer^KD^ mice with tissue acquisition 3 hours later revealed a significant increase in numbers of total BALF cells in the Mer^KD^ mice (Fig 4, *A*). Numbers of total BALF cells in WT mice 3 hours after apoptotic cell administration were near identical to those of mice that had been administered PBS as a control, indicating successful clearance of dead cells in the presence of intact Mer-mediated efferocytosis (Fig 4, *A*). In contrast, increased total numbers of cells were observed in Mer^KD^ mice, suggesting that the absence of Mer-mediated efferocytosis resulted in either failed clearance of the administered apoptotic cells, recruitment of inflammatory cells in response to the apoptotic cells, or both. Indeed, AMs (CD45^+^CD11c^+^ cells; see Fig E6, *A*, in this article's Online Repository at www.jacionline.org) from Mer^KD^ mice were characterized by reduced phagocytosis of the administered apoptotic cells *in vivo* (Fig 4, *B* and *C*). Minimal phagocytosis was observed in lung interstitial macrophages, which also express Mer, from either WT or Mer^KD^ mice. This is consistent with their limited anatomic ability to access the airway lumen (data not shown).^29^

In addition, an increased proportion of the administered apoptotic cells (mouse CD45^−^CellTracker^+^) was recovered in BALF from Mer^KD^ mice (Fig 4, *D*), highlighting the importance of Mer in mediating apoptotic cell clearance within the airway lumen. In parallel, an increased percentage of mouse neutrophils (CD45^+^Ly6G^+^ cells) was observed in BALF from Mer^KD^ mice (Fig 4, *E*). This increase in neutrophil counts was not observed in BALF recovered from Mer^KD^ mice immediately after administration of apoptotic cells (0 hours after intratracheal apoptotic cells), confirming that neutrophils were recruited in a time-dependent fashion specifically in the Mer^KD^ mice in response to apoptotic cells (Fig 4, *E*).

Lastly, a population of low SSC/FSC events that did not express markers of AM or epithelial cell origin (epithelial cell adhesion molecule–negative CD11c^−^F4/80^−^) was present in BALF from Mer^KD^ mice at 3 hours after intratracheal apoptotic cells. This population was minimal in the WT mice (Fig 4, *F*, and see Fig E6, *B* and *C*) at 3 hours and minimal at 0 hours in the Mer^KD^ mice (data not shown). Because uncleared apoptotic cells can undergo necrosis and release damage-associated molecular patterns, leading to recruitment of inflammatory cells,^30^ we hypothesized that the low SSC/FSC events were necrotic debris from the instilled human granulocytes. Consistent with this, cytocentrifuge preparations of flow-sorted low-FSC/SSC events revealed only cellular debris in comparison with sorted CD45^+^F4/80^+^CD11c^+^ events, which clearly demarcated the AM population (see Fig E6, *D* and *E*). Overall, these data demonstrate that Mer augments apoptotic cell clearance *in vivo* to dampen inflammation in response to dying cells.

## Discussion

Restoration of tissue homeostasis after tissue injury or infection requires termination of proinflammatory signaling and resolution of the inflammatory response. Control of the resolution process is achieved through a combination of local production of proresolution mediators and apoptosis of recruited inflammatory cells, together with their phagocytic removal.^2^, ^31^ It is widely accepted that disruption of the processes underlying the timely resolution of inflammation represents a significant contributory factor to the development of many inflammatory diseases. One corollary of the pivotal role of dysregulated resolution of inflammation in disease pathogenesis is that pharmacologic modulation of the processes underlying inflammation resolution represents an attractive strategy to attenuate ongoing inflammation and accelerate restoration of tissue homeostasis.^32^, ^33^ In support of this suggestion, induction of apoptosis of either neutrophils or eosinophils in mouse models of sterile, infectious, or allergic inflammation results in reduced inflammation in the airways and accelerated resolution of inflammation.^9^, ^18^, ^34^

Induction of granulocyte apoptosis during inflammation would exert beneficial effects by directly reducing the overall tissue burden of granulocytes and by limiting the release of cellular contents that contribute to further tissue damage and the development of persistent inflammation. Moreover, there might be additional indirect effects as a consequence of phagocyte uptake of apoptotic cells. Both “professional” and “nonprofessional” phagocytes, including airway epithelial cells, can mediate apoptotic cell clearance through multiple molecular pathways.^4^, ^35^ Such functional redundancy is thought to reflect the importance of effective apoptotic cell removal in both homeostatic and inflammatory processes.

Efferocytosis promotes the resolution process through modulation of phagocyte production and release of anti-inflammatory lipids and cytokines together with suppression of proinflammatory cytokine release.^36^, ^37^ In particular, the receptor tyrosine kinases Axl and Mer mediate clearance of apoptotic cells and membranes by dendritic cells and macrophages. Axl and Mer exhibit segregation in terms of both expression and activity in a variety of tissue settings, suggesting that they can perform distinct yet complementary physiologic roles.^12^ Expression of Axl is strongly induced by Toll-like receptor ligands and has been shown to play a major role in immunosuppression during inflammation. In contrast, Mer is upregulated by liver X receptor ligands and glucocorticoids and is thought to function predominantly in tissue homeostasis. However, antibody-mediated inhibition of Mer exacerbates inflammation after LPS challenge in the lung, and augmentation of Mer activity exerts protective effects.^16^, ^38^ Together with evidence that resolution-phase macrophages express high levels of Mer, these data suggest that Mer represents an important contributor to the process by which inflammation normally resolves.

In this study we have examined the role of apoptotic cell clearance in the resolution of inflammation associated with airway allergy. We report a number of novel findings that extend our understanding of the role of apoptotic cell clearance and Mer-mediated signaling in inflammation and tissue repair. First, we have demonstrated that onset of inflammation in response to OVA challenge in the lung is similar in the absence of Mer, with equivalent numbers of eosinophils present in the BALF of WT and Mer^KD^ mice at day 3. However, at later time points (day 7), BALF eosinophils persist in the Mer^KD^ mice, together with increased BALF protein levels. The presence of ongoing inflammation in the absence of Mer demonstrates that Mer is an important contributor to the efficiency of resolving eosinophilic inflammation in the airways. Yet the cellular inflammation in Mer^KD^ mice challenged with OVA returned toward baseline levels by day 10, suggesting that Mer-independent mechanisms ultimately allow clearance of recruited eosinophils in Mer^KD^ mice. The lack of an effect of loss of Mer on lung histology and on numbers of tissue eosinophils might be due to different mechanisms involved in eosinophil clearance in the airways and in the interstitial regions. One possibility is that eosinophils exhibit differential susceptibility to apoptosis in these distinct microenvironments, with airway eosinophils being more sensitive to undergoing pharmacologic induction of apoptosis and subsequent phagocytic clearance than interstitial eosinophils.^9^

Second, contrary to expectation, we did not observe highly increased proinflammatory cytokine profiles in Mer^KD^ mice at any of the time points examined during the course of the OVA-induced inflammatory response. Although Mer has been reported to suppress macrophage TNF production (eg, after LPS-induced inflammation in the peritoneal cavity or in the lung^26^), it is possible that there might be stimulus-specific effects and that Mer does not act to counterregulate a T_H_2-mediated inflammatory response. Comparison of expression levels at day 7 revealed that some potentially important chemokines, such as CCL12 and CCL5 (RANTES), which can act to recruit eosinophils, were present at increased levels in Mer^KD^ mice. However, these chemokines were present at relatively low levels in both WT and Mer^KD^ mice. Similarly, CCL11 and IL-5, which are important for eosinophil recruitment and survival, were expressed at roughly equivalent or lower levels in Mer^KD^ mice at 7 days. We suggest that these changes in chemokine/cytokine profiles in the Mer^KD^ mice are unlikely to account for the significant differences in eosinophil numbers observed. Whether Mer-driven resolution of allergic inflammation is associated with changes in the production of proresolving lipid mediators remains to be determined, but in a model of sterile peritonitis, Mer deficiency was associated with reduced levels of lipoxin A4 and resolvin D1.^39^

Third, the delayed resolution of inflammation we observed in Mer^KD^ mice was accompanied by increased airway resistance, suggesting that the altered inflammatory response in the absence of Mer has consequences in terms of lung function. Because airway resistance was similar in naive WT and Mer^KD^ animals, Mer is unlikely to represent a dominant factor in regulating airway function under homeostatic conditions. However, perturbation of lung homeostasis after injury or infection could highlight the role for Mer in regulation of responses to airway challenge. We did not find any significant changes in airway mucus production between WT and Mer^KD^ mice. It is possible that the differences in airway inflammation and resistance we observed do not affect mucus production or that mucus production is a less sensitive indicator of altered inflammation resolution.

To investigate the underlying mechanism of Mer in the process of inflammation resolution, we tested directly whether induction of high levels of apoptosis in eosinophils would reveal differences in the capacity for clearance of apoptotic cells in Mer^KD^ mice. In these experiments we treated animals with dexamethasone at the peak of BALF eosinophil recruitment and tracked the extent of apoptosis present in BALF. We observed approximately twice as many Annexin V^+^ apoptotic eosinophils in Mer^KD^ mice when compared with WT mice, which is consistent with a compromised capacity for eosinophil clearance in the absence of Mer. Glucocorticoids also act to increase Mer expression and function in macrophages, which would further highlight the effect of Mer deficiency in this experimental model. Although our experiments did not specifically examine the possibility that some dexamethasone-treated eosinophils were progressing directly to necrosis *in vivo* without having first undergone apoptosis (primary necrosis), this represents an important area for future study. Eosinophils activated by inflammatory mediators can undergo primary necrosis more readily,^40^, ^41^ and our subsequent experiments demonstrate that apoptotic cells lose their membrane integrity *in vivo* to become necrotic (secondary necrosis^42^), with this effect being marked in the absence of Mer-dependent cell clearance. Our data demonstrate that approximately 25% to 30% of the total capacity for apoptotic cell clearance of BMDMs or AMs is Mer dependent. Assuming that Mer mediates a similar proportion of macrophage capacity *in vivo*, the extent of apoptosis occurring during resolution of an inflammatory response might be sufficient to overwhelm the Mer-independent phagocytosis component, leading to enhanced necrosis and amplification of inflammation.

In this article we identified a role for apoptotic cell clearance by Mer in the setting of allergic airway inflammation, demonstrating a delay in resolution of inflammation in Mer^KD^ mice. Together, our data demonstrate that apoptotic cell clearance by phagocytes contributes directly to the resolution of allergic airway inflammation, suggesting augmentation of apoptotic cell clearance as a potential therapeutic strategy for treating allergic inflammation in human subjects.

**Key messages**

• Mer drives clearance of apoptotic eosinophils, key cells in allergic airway inflammation.
• Absence of Mer leads to delayed resolution of inflammation and increased airway resistance in allergic airway inflammation.
• Therefore augmenting apoptotic cell clearance is a potential therapeutic strategy for treating allergic inflammation.
